# Effectiveness of Electroencephalographic Neurofeedback for Parkinson’s Disease: A Systematic Review and Meta-Analysis

**DOI:** 10.3390/jcm14196929

**Published:** 2025-09-30

**Authors:** Leon Andreas W. R. von Altdorf, Martyn Bracewell, Andrew Cooke

**Affiliations:** 1 College of Medicine and Health, Bangor University, Gwynedd LL57 2DG, UK; 2 North Wales Medical School, Bangor University, Gwynedd LL57 2DG, UK; 3 Walton Centre NHS Foundation Trust, Liverpool L9 7LJ, UK; 4 Institute for the Psychology of Elite Performance (IPEP), Bangor University, Gwynedd LL57 2DG, UK

**Keywords:** EEG, biofeedback, motor symptoms, cortical activity, neuromodulation, neurorehabilitation, brain–computer interface, non-pharmacological, non-invasive therapy

## Abstract

**Background:** Electroencephalographic (EEG) neurofeedback training is gaining traction as a non-pharmacological treatment option for Parkinson’s disease (PD). This paper reports the first pre-registered, integrated systematic review and meta-analysis of studies examining the effects of EEG neurofeedback on cortical activity and motor function in people with PD. **Method:** We searched Cochrane Databases, PubMed, Embase, Scopus, Web of Science, PsycInfo, grey literature repositories, and trial registers for EEG neurofeedback studies in people with PD. We included randomized controlled trials, single-group experiments, and case studies. We assessed risk of bias using the Cochrane Risk of Bias 2 and Risk of Bias in Non-Randomized Studies tools, and we used the Grading of Recommendations, Assessment, Development and Evaluations tool to assess certainty in the evidence and resultant interpretations. Random-effects meta-analyses were performed. **Results:** A total of 11 studies (143 participants; Hoehn and Yahr I–IV) met the criteria for inclusion. A first meta-analysis revealed that EEG activity is modified in the prescribed way by neurofeedback interventions. The effect size is large (SMD = 1.30, 95% CI = 0.50–2.10, *p* = 0.001). Certainty in the estimate is high. Despite successful cortical modulation, a subsequent meta-analysis revealed inconclusive effects of EEG neurofeedback on motor symptomology. The effect size is small (SMD = 0.10, 95% CI = −1.03–1.23, *p* = 0.86). Certainty in the estimates is low. Narrative evidence revealed that interventions are well-received and may yield specific benefits not detected by general symptomology reports. **Conclusion:** EEG neurofeedback successfully modulates cortical activity in people with PD, but downstream impacts on motor function remain unclear. The neuromodulatory potential of EEG neurofeedback in people with PD is encouraging. Additional well-powered and high-quality research into the effects of EEG neurofeedback in PD is warranted.

## 1. Introduction

Parkinson’s disease (PD) is the second most common neurodegenerative disease, after Alzheimer’s disease, affecting 4% of the global population aged over 80 years [1]. The cardinal symptoms of PD include varying degrees of hypokinesia, bradykinesia, rigidity, and resting tremor, as well as non-motor symptoms, such as progressive executive dysfunction. They have an adverse impact on quality of life [2,3] and often demand an increasing need for care, placing strain on public health systems and emphasizing the pressing need for effective management and treatment strategies.

The pathogenesis of PD is characterized by a progressive loss of dopaminergic neurons in the substantia nigra [1], and the subsequent loss of dopamine is responsible for dysregulated movement, memory, and allied physiological processes that characterize the symptoms of PD [4]. The main treatment options for PD are pharmacological, such as levodopa, dopamine agonists, cathechol–o–methyl transferase inhibitors, monoamine oxidase B inhibitors, and anticholinergics, where each of these agents is designed to act on the neurotransmission dysregulation [5]. There are also surgical treatment options, such as electrode implantation to brain regions, such as the subthalamic nucleus, to allow for externally regulated deep brain stimulation [6]. However, pharmacological treatment options are blighted by side effects, including dyskinesias, confusion, and hallucinations [7], while surgical treatment brings risks of complications during and after implantation (e.g., bleeding and infection) [8]. Accordingly, there is a need for non-pharmacological and non-surgical alternative or adjunct treatment options. One potential option gaining increasing research attention is electroencephalographic (EEG) neurofeedback.

### 1.1. EEG Neurofeedback

EEG neurofeedback uses sensors to record electrical activity on the scalp paired with software to translate the signals into specified features of cortical activity in near real time [9]. Cortical activity is fed back to participants, typically via visual displays or auditory tones that offer positive reinforcement to help individuals self-regulate, with the goal of producing patterns of activity thought to be conducive to cognition or action [10]. Studies of non-clinical populations have demonstrated positive effects of neurofeedback training on cortical self-regulation, motor control, and motor performance (e.g., [11,12,13]). For example, approximately 4 h of neurofeedback training allowed a sample of experienced golfers to increase the spectral power of their pre-movement sensorimotor rhythm (12–15 Hz), and this was associated with enhanced golf putting accuracy compared to non-neurofeedback control participants [11]. EEG neurofeedback interventions are therefore ripe for exploration in clinical populations where the regulation of aberrant cortical activity may enhance motor function [14].

### 1.2. EEG Neurofeedback for Parkinson’s Disease

Modern EEG neurofeedback equipment is portable, minimally invasive to wear, and much cheaper to procure and operate than alternative neurofeedback modalities (e.g., fMRI), and in the majority of cases, EEG neurofeedback has no side effects [15]. It could thereby represent a valuable, convenient, and cost-effective non-pharmacological treatment option for people with PD. However, the effectiveness of neurofeedback training hinges on identifying and targeting features of cortical activity closely associated with the desired motor functions [14]. EEG studies of people with PD have consistently revealed impaired pre-movement event-related desynchronization in the alpha and mu EEG frequency bands (around 8–12 Hz) or reduced negativity of pre-movement EEG slow waves, such as the Bereitschaftspotential (readiness potential), when compared to age-matched controls (e.g., [16,17]). These aberrant cortical patterns may be associated with imprecise pre-programming of initial movement commands, delayed movement initiation, and, in extreme cases, akinesia [18]. Research has also revealed excessive sensorimotor rhythm (SMR—around 12–15 Hz) and beta (around 13–30 Hz) power at rest and during the preparation and execution of actions in people with PD, with these EEG features being further associated with bradykinesia and tremor [19,20]. Accordingly, there is a strong mechanistic case for EEG neurofeedback interventions targeting the modulation of any one or combination of the readiness potential and EEG alpha, mu, SMR, and beta spectral power, with the goal of improving motor function in people with PD.

### 1.3. The Present Review and Meta-Analysis: Aims and Objectives

Given the rising prevalence of PD, the limitations of pharmacological treatments, and the enticing potential of neurofeedback treatment options, a rigorous review to evaluate the effects of neurofeedback in PD is warranted. Seminal case studies of EEG neurofeedback applied to people with PD emerged in the 2000s (e.g., [21]), and there has been a slow but increasing rate of studies published since then. Three existing reviews have summarized the research to date [22,23,24], although only the most recent review (i.e., [24]) focused specifically on EEG neurofeedback. All three noted mixed findings and concluded that neurofeedback may hold promise, but none conducted meta-analyses to provide a data-driven synthesis.

Thus, despite increasing interest, it remains unclear whether EEG-based neurofeedback can reliably modulate cortical activity and improve motor symptoms in PD. Importantly, no systematic review to date has quantitatively pooled evidence on EEG neurofeedback in this population. To address this gap, this article asks the following question: Does EEG neurofeedback promote targeted modulation of cortical activity and motor outcomes in people with PD? By conducting the first systematic review and meta-analysis devoted to EEG neurofeedback in PD, this study advances the field beyond narrative-only reviews.

### 1.4. Primary Objectives

1. Determine whether EEG neurofeedback promotes targeted modulation of EEG activity in people with PD.
2. Determine the effects of EEG neurofeedback interventions on motor function/symptomology in people with PD.

These objectives are assessed by meta-analyses and a narrative review of systematically identified studies employing EEG neurofeedback interventions for people with PD.

### 1.5. Secondary Objectives

1. Provide a narrative discussion of EEG neurofeedback studies in people with PD, considering features such as risk of bias, certainty of evidence, neurofeedback protocol used, and EEG and motor outcome measures, to aid the interpretation of the meta-analytical results.
2. Identify targeted avenues emerging from the literature for future EEG neurofeedback research.

## 2. Materials and Methods

Our integrated systematic review and meta-analysis was conducted in accordance with the Preferred Reporting Items for Systematic reviews and Meta-Analyses (PRISMA; [25]) checklist and the Cochrane Handbook for systematic reviews of interventions (v6.5) [26]. Our protocol was pre-registered on the National Institute for Health and Care Research PROSPERO review register (https://www.crd.york.ac.uk/PROSPERO/view/CRD420251059697 (accessed on 10 June 2025)).

### 2.1. Selection Criteria

We focused on prospective EEG neurofeedback intervention studies in adults with a clinical diagnosis of PD [27]. The included interventions consisted of EEG neurofeedback alone or alongside standard treatments, compared to no treatment, placebo, waiting list, or standard care. Studies reported EEG activity and/or motor outcomes as primary measures, with secondary outcomes (e.g., non-motor symptoms and quality of life) considered in the narrative synthesis. The full inclusion criteria are summarized in Table 1.

### 2.2. Search Strategy

We used the following healthcare electronic databases and clinical trial registers to conduct our literature search: Cochrane Database of Systematic Reviews, Cochrane Database of Abstracts of Effectiveness, CENTRAL via The Cochrane Register of Studies, PubMed, Embase, Scopus, Web of Science Core Collection, PsycInfo, the WHO International Clinical Trials Registry Platform, ClinicalTrials.gov, EUDRACT, and ISRCTN. We also searched the UK National Research Register to identify archived studies up until 2021, when the register closed. To maximize rigor, we additionally searched the “System for Information on Grey Literature” for bibliographical references, doctoral dissertations, conference papers, research reports, and other types of grey literature. We searched the ‘Research Registry’ (an IJS/Wolters Kluwer resource), the NIHR “Be Part of Research”, and the HRA Research Summaries for ongoing studies that had not yet reached the publication stage but may have reported interim results. We also performed lateral searches, such as checking reference lists and tracking citations, as recommended by [28]. The specific search filters employed for each database are provided in the Supplementary Materials. Three separate searches were performed alongside a specialist librarian, with the most recent one on 24 October 2024.

### 2.3. Study Selection

**Figure 1:**
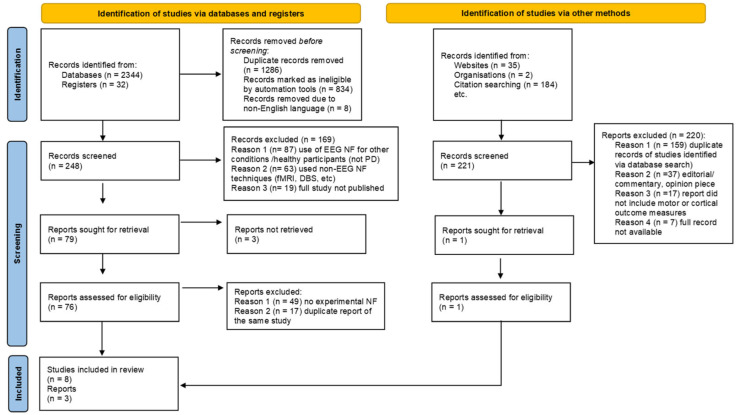
PRISMA 2020 flow diagram for new systematic reviews that included searches of databases, registers, and other sources.

The first author screened the results and removed articles that did not meet the inclusion criteria. The Ryyan web resource [29] was used to automate deduplication, filtering, text mining, and relevance ranking. Two reviewers then independently assessed the titles and abstracts of all records identified, and disagreements were resolved by discussion. Studies that did not meet the inclusion criteria were excluded. The full text resource was obtained for potentially relevant studies. Studies were not anonymized before assessment. The status of identified studies is reported using the PRISMA flow chart [25] (see Figure 1), as recommended by [26].

### 2.4. Data Extraction and Management

A standardized pre-defined data extraction form was used to capture relevant data spanning the study characteristics, quality assessments, participants, interventions, methods, and results. A note section was added to facilitate discussion. The study characteristics documented were author(s), title, and publication data. Data collected in relation to participants included the demographics (age, gender, diagnostic criteria, severity of PD, and severity of motor impairment) and sample characteristics (total number of participants (per group if relevant) in whom the outcomes were measured (means and SD)). Data collected in relation to methods included the study design, setting, duration, group allocation methods or randomization (if relevant), intervention crossover, length of follow-up, and time of outcome assessment. Data related to the interventions comprised the mode of EEG neurofeedback, type of control or combined intervention, duration, and frequency. The quality assessment data captured were allocation concealment, randomization sequence generation, blinding, outcome data completeness, outcome reporting, and other sources of bias. Primary outcomes collected were EEG activity and measures of motor function. Secondary outcomes were non-motor symptoms, quality of life, activities of daily living, indices of intervention efficacy, or acceptability. We recorded differences in the outcome variables between intervention and control groups and/or changes from baseline/pre-test to post-test or across other timepoints, where reported. This process was initially completed by the first author and then presented to the other authors, who engaged in a check-and-challenge discussion until consensus was reached.

### 2.5. Risk of Bias Assessment

We assessed the risk of bias and evaluated the methodology of studies included in the review, in accordance with [26]. Specifically, we evaluated the randomization process, any deviations from planned interventions, the completeness of the outcome data, selective reporting, and measurement bias using the standard version of the RoB 2 tool (Cochrane Risk of Bias in randomized trials) [30] and the RoBINS-I tool (V2) (Risk of Bias in non-randomized Studies/interventions) [31] to assess risk of bias in randomized and non-randomized studies, respectively. The output was visualized using Robvis—the visualization tool for risk of bias assessments in a systematic review [32].

### 2.6. Meta-Analysis

In addition to the narrative review, we sought to perform meta-analyses of studies reporting the requisite data in order to establish, in more objective terms, the impact of EEG neurofeedback interventions on EEG activity and on motor symptoms in people with PD. We conducted the review and meta-analyses via Cochrane RevMan Ò (v 6.5) [26]. We used a random-effects meta-analysis since included studies estimated different effect sizes and to account for both study variance (due to sampling errors) and between-study variance due to the heterogeneity of the interventions.

We converted all effect estimates into standardized mean differences (SMDs) and conducted two sets of analyses stratified by study design. For randomized controlled trials (RCTs), we performed meta-analyses on the between-group SMDs post-test (intervention vs. control) to estimate the causal effect of the intervention. For studies without a control group (as well as the intervention arms of RCTs), we performed meta-analyses on the within-group pre-intervention to post-intervention SMDs to provide non-causal estimates of change over time in intervention participants.

Analyses were conducted separately for motor symptoms and EEG outcomes. For the motor symptom meta-analysis, we included studies reporting Unified Parkinson’s Disease Rating Scale Part III scores (UPDRS III) [33]. EEG measures in the cortical activity meta-analysis were more heterogeneous, as studies targeted different neural features (e.g., sensorimotor rhythm and beta power). Because our primary interest was whether interventions altered the specific EEG feature each study aimed to modulate, we used the standardized mean difference (SMD) to harmonize these diverse outcomes into a single construct of intervention-related cortical modulation.

Studies were weighted using the inverse-variance method, meaning that studies with larger sample sizes and more precise effect estimates contributed proportionally more to the pooled estimate, while the random-effects model also incorporated between-study variance (τ^2^) to help ensure that studies were weighted more equally than in fixed-effect models.

We report SMDs with 95% confidence intervals, where values of 0.2, 0.5, and 0.8 can be interpreted as small, medium, and large effects [34]. Heterogeneity was assessed using the I^2^ statistic, with values < 30% indicating low heterogeneity, 30–50% indicating moderate heterogeneity, and >50% indicating high heterogeneity.

### 2.7. Subgroup Analysis

Subgroup analyses via characteristics that might be expected to impact the outcomes, such as the number and duration of neurofeedback sessions, were considered, but due to the limited number of identified studies, we did not proceed with formal analyses. These features were instead considered in our narrative review.

### 2.8. Assessment of Reporting Bias (Certainty of Evidence)

We used the Grading of Recommendations, Assessment, Development and Evaluation (GRADE) approach to assess the certainty of evidence for studies included in the meta-analyses. The term ‘certainty of evidence’ conveys the confidence in the estimates of the effect and indicates how likely the findings are to be correct. This does not relate directly to the quality of the studies themselves, but rather to the confidence in the findings for a specific outcome in this study. It specifies high, moderate, low, or very low certainty for each outcome, where high reflects high confidence that the presented estimate of effects lies close to the true effect, and very low reflects little confidence in the estimate, acknowledging that the true effect is likely to be substantially different. Estimates afforded very low ratings tend to originate from studies deemed imprecise, indirect, or deemed to have a high risk of bias.

We established GRADE assessments of certainty by evaluating the following five domains [35]:

1. Risk of bias: evaluated using version 2 of the Cochrane risk of bias tool for randomized trials (RoB 2 for the randomized parallel group or crossover trials and ROBINS-I for non-randomized studies of an intervention). See the risk of bias section above for more information.
2. Inconsistency: evaluated by examining the variability in findings across studies.
3. Indirectness: evaluated by establishing whether the evidence directly addresses the target population (PD patients) and intervention (EEG neurofeedback).
4. Imprecision: evaluation of the precision of the effect estimates.
5. Publication bias: the potential for studies with positive results to have a higher likelihood of publication.

Tools such as the risk of bias assessments and data such as the confidence intervals and the heterogeneity of effects within and across studies informed our GRADE assessments for risk of bias, inconsistency, and imprecision. Our targeted search strategy generally ensured high certainty of the directness/relevance of the findings to our target population and research questions. Publication bias was assessed by discussion due to an insufficient number of randomized controlled studies to perform an objective assessment/draw meaningful funnel plots [36]. While evidence certainty is downgraded for high scores on any of the five factors listed above, GRADE also recommends upgrading evidence certainty where the following conditions apply:

1. Large and/or consistent effect sizes
2. A dose-response gradient
3. Positive effects despite plausible confounds

These factors were graded on a study-by-study basis and across pools of studies and discussed by the team to reach a consensus.

## 3. Results

### 3.1. Search Results

The systematic literature search yielded 2597 records. After removing duplicates, non-English reports, and ineligible studies, 469 records were screened, and 77 full texts were assessed for eligibility. Following exclusions (e.g., no EEG neurofeedback group and duplicate reports), 11 studies, involving 143 participants, were identified for inclusion in the review. The full study selection process is illustrated in Figure 1.

### 3.2. Included Studies

Summary details of the 11 included studies are presented in Table 2 and Table 3. In brief, the included studies comprised five randomized controlled trials, two non-randomized/within-subject neurofeedback experiments, and four neurofeedback case reports/studies. While all used EEG neurofeedback, eight reported EEG data as an outcome, ten reported some form of motor symptoms as an outcome, and five additionally reported non-motor outcomes (e.g., anxiety, depression, quality of life, and intervention acceptability). Data from all included studies contributed to the narrative review, and those reporting the requisite data were additionally included in our meta-analyses.

### 3.3. Study Quality Appraisal

The risk of bias assessment for randomized controlled trials (RoB 2) and non-randomized studies (ROBINS-I) indicated that all studies had a moderate risk of bias in at least one of the assessed domains. Overall, the randomized controlled trials were deemed to be at a moderate risk of bias. All but one study [40] were deemed to have a low risk of bias due to deviations from the intended intervention, but only one study [45] had a low risk of bias arising from the randomization process—randomization was rarely double-blinded. Potential bias due to missing outcome data, measurement of the outcome, and selection of the reported result varied across studies.

**Figure 2:**
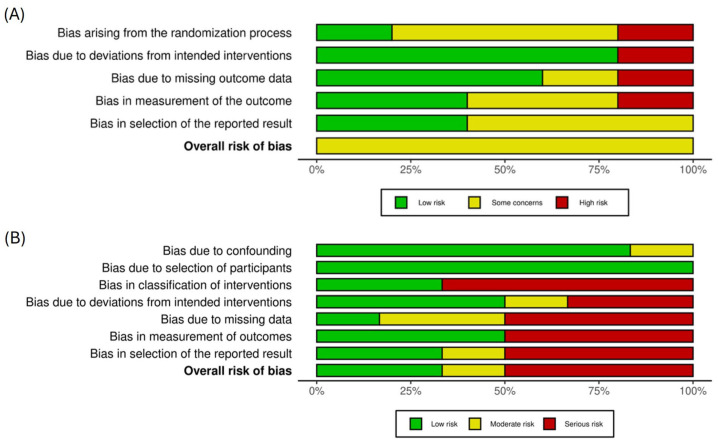
(**A**) Risk of bias assessment in randomized controlled trials. (**B**) Risk of bias assessment in non-randomized studies.

Of the non-randomized studies, three were classified as having a relatively high risk of bias [21,43,44], one had a moderate risk of bias [38], and two had a low risk of bias [39,41]. All studies were considered to have a low risk of bias in their participant selection, but there was a general moderate to high risk of bias across the studies due to missing data. Bias risk tended to be higher in the case studies (e.g., [21,43,44]). Case studies tended to provide more narrative/observational accounts of intervention effects on brain and behavior, whereas the experimental and randomized controlled trials tended to offer a fuller report of their methods and data. A summary of the risk of bias outcomes is presented in Figure 2. Study-level outcomes are summarized in Table 2 and are fully available in the Supplementary Materials.

### 3.4. Meta-Analyses

**Figure 3:**
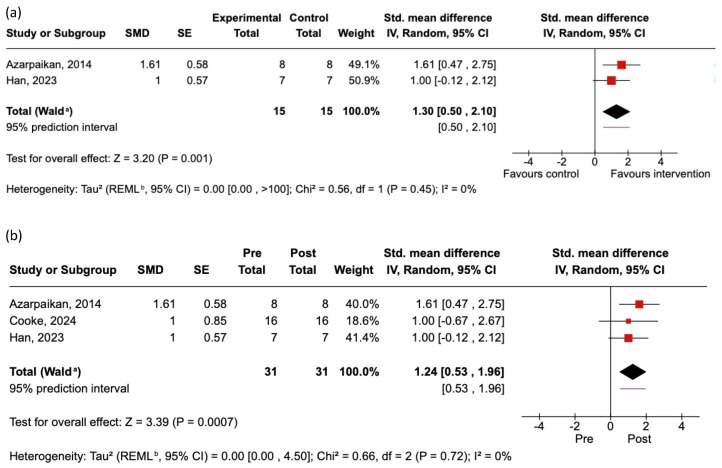
Summary of included studies and forest plots reporting the effects of neurofeedback on target EEG activity. The size of each square reflects the study weight in the random-effects model. The diamond represents the pooled effect estimate: (**a**) Meta-analysis of RCTs showing the SMD of the difference between the intervention group and the control group post-test. Positive scores reflect a difference in the targeted EEG feature between the intervention group and the control group post-test. (**b**) Meta-analysis of studies without a control group (as well as the intervention arms of RCTs) showing the SMD of the difference between pre-test and post-test. Positive scores reflect a change in the targeted EEG feature from pre-test to post-test among participants receiving neurofeedback interventions. ^a^ indicates CI calculated by Wald-type method. ^b^ indicates Tau^2^ calculated by Restricted Maximum-Likelihood method.

**Figure 4:**
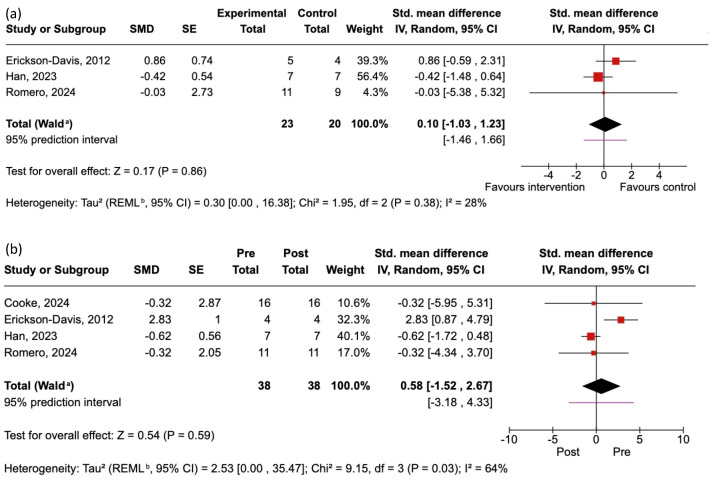
Summary of included studies and forest plots reporting the effects of neurofeedback on target UPDRS III assessed motor symptomology. The size of each square reflects the study weight in the random-effects model. The diamond represents the pooled effect estimate: (**a**) Meta-analysis of RCTs showing the SMD of the difference between the intervention group and the control group post-test. Negative scores reflect less severe motor symptomology in the intervention group than the control group post-test. (**b**) Meta-analysis of studies without a control group (as well as the intervention arms of RCTs) showing the SMD of the difference between pre-test and post-test. Negative scores reflect an improvement (i.e., reduced severity) in motor symptomology from pre-test to post-test among participants receiving neurofeedback interventions. ^a^ indicates CI calculated by Wald-type method. ^b^ indicates Tau^2^ calculated by Restricted Maximum-Likelihood method.

We performed separate meta-analyses to examine the effects of the identified EEG neurofeedback interventions on cortical activity and UPDRS Part III. Our meta-analytical findings are summarized below and in Figure 3 and Figure 4.

### 3.5. Effects of EEG Neurofeedback on Cortical Activity

To test whether EEG neurofeedback successfully modulated cortical activity in the prescribed way, we used the SMD in the EEG neurofeedback target identified by each RCT between the intervention group and the control group post-test. Two studies provided the requisite EEG data for inclusion (Shi et al. (2023) [46] also provided requisite data, but it was excluded because their study and that by Han et al. (2023) [42] shared the same neurofeedback participants). The findings are summarized in Figure 3a. There was a large significant effect in favor of the effectiveness of the neurofeedback interventions, causing the targeted cortical activity changes.

For studies without a control group (as well as the intervention arms of RCTs), we performed meta-analyses on the within-group pre-intervention to post-intervention SMDs. Three studies provided the requisite EEG data for inclusion. The findings are summarized in Figure 3b. There was a large significant effect in favor of the prescribed changes in cortical activity from pre-tests to post-tests among intervention group participants.

While the number of studies included in the analyses is low, the relatively large and highly consistent effects across the assessed studies yielded a high certainty of evidence for neurofeedback-related changes in cortical activity among people with PD according to the GRADE criteria.

### 3.6. Effects of EEG Neurofeedback on Motor Symptoms Assessed by UPDRS III

Two random-effect meta-analyses were also performed to test the impact of EEG neurofeedback on motor symptoms of PD. For RCTs, we used the SMD in the UPDRS Part III motor assessment between the intervention group and the control group post-test. Three studies provided the requisite UPDRS III data for inclusion. The findings are summarized in Figure 4a. There was a small and non-significant effect, providing no conclusive evidence of EEG neurofeedback impacting UPDRS III assessed motor symptoms.

For studies without a control group (as well as the intervention arms of RCTs), we performed meta-analyses on the within-group pre-intervention to post-intervention SMDs. Four studies provided the requisite UPDRS III data for inclusion. The findings are summarized in Figure 4b. There was a small and non-significant effect, providing no conclusive evidence of changes in the motor symptoms of PD from pre-tests to post-tests among intervention group participants.

Due to a low number of studies, combined with inconsistent effects (moderate to high heterogeneity of variance) across studies, the certainty of evidence for EEG neurofeedback effects on motor symptoms of PD was deemed to be low according to the GRADE criteria.

Please note that, while illustrative, all our meta-analytic outcomes should be interpreted with a degree of caution based on relatively small samples and the moderate risk of bias concerns (especially around blinding and selective reporting) documented in Table 2, as well as the risk of bias assessments (Figure 2 and Supplementary Materials).

## 4. Discussion

This paper provides the first systematic review and meta-analysis targeted specifically on the effects of EEG neurofeedback as a non-pharmacological treatment for PD. We add a narrative to the meta-analytic findings to help interpret the results and identify avenues for future research and applied practice.

### 4.1. Effects of EEG Neurofeedback on Cortical Activity

Our first objective was to determine whether EEG neurofeedback can promote the prescribed changes in cortical activity in people with PD. Successful cortical modulation is necessary for neurofeedback interventions to have a chance at promoting the expected downstream changes in cognition/behavior via the hypothesized cortical pathways (i.e., beyond any benefits attributable to placebo). The results support successful cortical modulation via EEG neurofeedback. Our neuromodulation-focused meta-analyses revealed significant, consistent, and large effect sizes (e.g., neurofeedback interventions were associated with a 1.30 SD difference in targeted EEG activity compared to controls). This finding is tempered by the fact that only two studies and three studies contributed data to the RCT and within-group meta-analyses, respectively. Nevertheless, confidence in this conclusion is strengthened by the observation that all studies identified in the review—including those not included in the meta-analysis—reported some evidence of successful cortical modulation (Table 3).

It was noted in the case study by [43] that successful modulation by their single participant was achieved when she was ON but not when she was OFF her regular Parkinsonian medication. In addition, ref. [41] reported individual differences in responsiveness to their readiness potential neurofeedback protocol. Thus, while on average, the data provide very promising evidence in favor of EEG neurofeedback modulating cortical activity in people with PD, there are likely conditions or individual considerations that will allow some people with PD to respond more strongly than others. Notwithstanding, we conclude with regard to our first objective that the current evidence does endorse EEG neurofeedback as a valid treatment to modify aberrant cortical activity in people with PD.

Importantly, our confidence in the available evidence is graded as high, meaning that the true effect is likely to lie close to our meta-analytic estimate. This confidence is enhanced by the consistency of effects across studies despite potential confounders (e.g., variability in neurofeedback protocols across studies) that may have obscured outcomes. In brief, neurofeedback targets included a range of readiness potential-, alpha-, mu-, SMR-, and beta-related features across studies, with additional consideration of theta power in some of the studies (Table 3). They also ranged substantially in duration and frequency (session length: 9 min to 1 h; number of sessions: 2–24; total neurofeedback exposure: 18 min to 35 h (Table 3)). Potential moderators of the effect of neurofeedback interventions on cortical activity (e.g., neurofeedback feature targeted and total neurofeedback exposure) were not examined statistically here due to the relatively low number of studies. Narratively, there is no obvious evidence to indicate that a particular EEG feature was more successfully modulated than others. However, there is some narrative evidence that the amount of neurofeedback exposure is important. The two studies providing less than 1 h of total neurofeedback exposure were the only ones to report limited neuromodulation in some individuals [41] or conditions (i.e., when OFF medication) [43].

As the literature grows, it will be possible for future meta-analyses to consider subgroup analyses of factors, such as neurofeedback target and exposure duration. Based on the current evidence, we encourage future EEG neurofeedback interventions to continue to consider any EEG neurofeedback target that is grounded in a strong theoretical/mechanistic framework associated with the cognitive or behavioral features of interest [14]. However, researchers are advised to plan at least 1 h of total neurofeedback exposure to provide a realistic chance for participants to learn self-regulation, and potentially much longer if participants are to be trained while OFF their pharmacological medication. Researchers may also consider the utility of specific instructions/strategies to help participants shape their EEG activity in the most relevant way (e.g., [47]).

### 4.2. Effects of EEG Neurofeedback on Motor Function

Having established a positive effect of EEG neurofeedback on cortical modulation in people with PD, our next objective was to determine whether the intervention yielded the hypothesized benefits to motor outcomes. In spite of studies generally revealing the expected EEG changes, our meta-analysis of studies examining the effects of neurofeedback on PD motor symptoms assessed via the UPDRS III revealed little evidence for beneficial effects of EEG neurofeedback on general PD motor symptomology (SMD = 0.10, *ns*). Estimated effects of EEG neurofeedback on UPDRS III scores range from small positive to moderate negative (i.e., inconclusive), and our confidence in the estimate is low, meaning the true effect may be substantially different from our estimate (i.e., around zero).

Narratively, ref. [45] provided some evidence of significant improvement in UPSRS III assessed motor symptoms after combining EEG and rTMS neurofeedback, but no evidence of the benefits of EEG neurofeedback on its own. Meanwhile, ref. [38] reported mixed evidence in their case study, with their single participant displaying an increase in the severity of their motor symptoms from pre- to post- neurofeedback session one (i.e., 3 point increase on UPDRS III), followed by a 5-point UPDRS III decrease (i.e., symptomology improvement) from pre- to post- neurofeedback session two. The other studies reported no significant changes—in brief, UPDRS III scores were marginally and non-significantly improved [42], remained the same [39], or were marginally and non-significantly worsened [40] from before to after the respective EEG neurofeedback interventions.

Beyond the variables included in the meta-analysis, it is worth noting that several of the included studies reported data on other motor outcomes. For example, ref. [37] reported that participants trained to increase beta-power outperformed control participants on both dynamic and static balance assessments. Adopting a similar neurofeedback intervention, ref. [42] also reported a trend for improvement in dynamic balance from pre-test to post-test in their neurofeedback participants. However, they did not outperform controls. A similar trend for improved static balance across multiple assessments was revealed by [45], although, once again, the effect occurred across both treatment and control participants. While these more recent studies do not fully replicate the findings of [37], the collection of positive-leaning effects over time can serve as encouragement for future research to continue to examine the effects of both EEG neurofeedback and mere exposure to balance tests on the balance and stability of people with PD.

The speed of movement initiation is another motor outcome of interest. While ref. [39] did not reveal any beneficial effects of the decreased EEG mu power neurofeedback protocol on general motor symptoms (UPDRS III), the authors did reveal suggestive evidence that their intervention expedited the initiation of their primary handgrip force production task. The rationale for their mu neurofeedback protocol was based on the literature linking mu ERD to enhanced movement initiation (e.g., [16,48]); therefore, it is possible that their result reflects a specific benefit of their protocol that was captured by a task that was most sensitive to detect it and that was missed by more general symptomology measures.

Similarly, while their overall UPDRS III results were mixed, ref. [38] reported that their single participant appeared to show marked improvements in rigidity and gait assessments after neurofeedback treatment, while [44] reported observing post-neurofeedback improvements in tremor, dysgraphia, and freezing of gait across their three case participants. These potentially specific and case-by-case benefits may reflect decreases in beta power targeted by both of those studies, helping to promote more normal movement-related beta rhythms to aid specific aspects of gait and writing where excessive beta activity may be particularly problematic [49].

Notwithstanding, it is important to note that these subtle and suggestive benefits to certain aspects of motor function cannot be causally attributed to EEG neurofeedback yet. Overall, the weight of the existing evidence indicates that there is currently no conclusive support for clinically beneficial effects of EEG neurofeedback on motor function in people with PD.

### 4.3. Other Effects of EEG Neurofeedback in People with Parkinson’s Disease

While it is beyond the primary scope of this review, it is also worth noting that some of the studies reported suggestive beneficial effects of their EEG neurofeedback interventions on non-motor symptoms. For example, ref. [45] provided some evidence that EEG neurofeedback was associated with a small reduction in self-reported depressive symptoms, while [46] reported that multimodal EEG neurofeedback, combined with cardiac and respiratory biofeedback, was associated with reduced anxiety and depression. However, in [46], there were no benefits for the group that received EEG neurofeedback alone, suggesting that the non-motor benefits were not principally driven by cortical changes. Importantly, assessments of participant acceptability conducted by [39] revealed that their home-based EEG neurofeedback intervention was extremely well received by participants. Given that the currently reviewed studies identified EEG targets and developed their neurofeedback protocols with the aim of aiding motor functions, we would not expect to see widespread non-motor benefits. However, given the proof that EEG neurofeedback interventions generally modulate cortical activity in people with PD, combined with evidence that the interventions are generally liked by participants, it would be fruitful for future research to examine neurofeedback protocols beyond those targeting motor function in people with PD. For instance, frontal alpha asymmetry EEG neurofeedback is theorized to boost mood and reduce symptoms of anxiety and depression (e.g., [50]) and may be particularly ripe for exploration in people with PD. Such future research could also benefit from the use of recently developed PD-specific and culturally specific non-motor assessments that go beyond the traditional non-motor scales (e.g., Beck Anxiety Inventory) to provide more targeted and precise assessments tailored to real-world clinical needs (e.g., [51,52]).

### 4.4. Limitations

We note that our systematic review and meta-analysis is constrained by limitations inherent to the reviewed literature. First, the literature revealed only eleven studies, with a total of 143 participants meeting the inclusion criteria. Second, most of the available studies were non-randomized experiments and case reports, with only five randomized controlled trials. In addition to the small sample sizes, several studies have a high risk of bias in various domains, potentially limiting their internal validity and increasing the potential for biased estimates of effect sizes. Unclear randomization and blinding further increase the possibility that performance or selection biases could have obscured the observed effects, resulting in generally low to moderate certainty of evidence. The meta-analytic effects and narrative conclusions of this review should be viewed with these constraints in mind. They nonetheless provide a helpful indication of the direction of effects in the extant literature, and they provide evidence of certainty in the positive effects of EEG neurofeedback on cortical modulation in PD.

### 4.5. Implications and Future Research Directions

While evidence pertaining to the effects of EEG neurofeedback on motor outcomes in people with PD remains inconclusive, we provide the first meta-analytic evidence that EEG neurofeedback modulates cortical activity in the expected way. Further evidence from our narrative review of studies reveals potential for specific case-by-case benefits of various neurofeedback interventions, as well as evidence that these interventions are well-tolerated and liked among people with PD. Given the pressing need for efficacious non-pharmacological interventions for people with PD, future high-quality studies of the effects of neurofeedback are warranted. We have already outlined several avenues for future enquiry in the preceding sections. In addition to those, we recommend that all future neurofeedback studies follow the CONSORT statement [53] to ensure that the study design, conduct, recording, and reporting are appropriate and adequately documented and that trials are pre-registered to help reduce risk of bias. The heterogeneity of neurofeedback target(s), protocols, and outcome measures is already evident in the literature; this is expected given the absence of standardized procedures in this young and experimental treatment field. However, diverse practices across labs make thorough reporting and properly powered trials critical to allow meaningful interpretation of results and to permit subgroup/moderation effects to be explored by the systematic reviews and meta-analyses in the future.

Above all, we believe the clinical EEG neurofeedback field is now at a critical junction. To reach the next developmental phase, there is a need for research to evolve beyond the (useful, but relatively small-scale) laboratory studies documented to date and shift towards larger randomized controlled trials and home-based testing. At the same time, further work is needed to evaluate the feasibility, costs, and infrastructure required to integrate EEG neurofeedback into PD care, in anticipation of potential evidence for its clinical benefits in the years ahead. We anticipate that a wave of larger-scale and high-quality EEG neurofeedback trials will provide new and more comprehensive answers to the questions posed by this review in the years and decades to come.

## 5. Conclusions

Our systematic review and meta-analysis of the effects of EEG neurofeedback training on cortical modulation and motor outcomes provides seminal meta-analytic evidence that EEG neurofeedback promotes successful modulation of cortical activity in people with PD. However, the research available to date does not provide conclusive evidence for downstream beneficial effects of EEG neurofeedback on PD motor symptoms. Both primary conclusions are based on a small number of studies that adopted a variety of protocols, each with its own methodological limitations and a relatively small sample size, along with some risk of bias. More rigorous and well-powered trials are needed for a more precise determination of the effectiveness of EEG neurofeedback for people with PD. While the current effects of neurofeedback on general symptomology are unclear, the high tolerability and promising neuromodulatory potential of EEG neurofeedback provide a compelling rationale for further research. 

## Figures and Tables

**Table 1:** Criteria for study selection.

Population (2)	Intervention (1)	Comparison (6)	Outcome (6)
Adult Parkinson’s disease	EEG Neurofeedback training	No intervention Standard care Withdrawal of medication (OFF/ON comparison) Placebo/Sham EEG Other forms of neurofeedback Multimodal interventions	EEG activity Motor symptom severity Non-motor symptom severity Quality of life and activities of daily living Efficacy/Clinical improvement Acceptability

Note: this table summarizes the Patient, Intervention, Outcome, and Comparison (PICO) framework elements used for selecting the studies included in the review.

**Table 2:** Overview of included studies.

Study Authors, (Publication Year), (Country), *Journal*	Study Aims	Study Design	Symptom/Activity Targeted	Sample Size	Control Intervention	Follow-Up	Outcomes (As Reported)	Overall Risk of Bias (RoB2/RoBINS)
Azarpaikan, Torbati, and Sohrabi (2014) (Iran) *Gait & Posture* [37]	Evaluate the effects of EEG NF on dynamic and static balance in people with PD.	RCT	Cortical activity: increased amplitude of low beta wave (12–15 Hz) and decreased amplitude of theta wave (4–7 Hz) Motor symptoms: static and dynamic balance	n = 16 Intervention: 8 PD (4M/4F); Control: 8 PD (4M/4F) *M*_age_ = 74.7 H&Y 2.35 (SD = 0.08)	Sham EEG NF	No	Cortical activity: theta wave amplitude increased and beta wave amplitude decreased across NF sessions in the NF group. Motor symptoms: significant improvements occurred in static and dynamic balance in the intervention group. Successful use of EEG NF to train people with PD to produce prescribed patterns of cortical activity, resulting in improved balance task performance	moderate
Cook, Pfeifer, and Tass (2021) (USA) *Frontiers in Neuroscience* [38]	Explore the effects of EEG NF on SMR power (rate of SMR burst) over the motor cortex.	Case study	Cortical activity: rate, duration, and amplitude of beta and SMR bursts, RP in the beta band General motor symptoms	n = 1 1 PD, ≈55 years old, 10 years since diagnosis, H&Y 2 plus 1 pilot patient, data not reported	No control intervention (assessments made ON and OFF medication)	No	Cortical activity: rate of SMR bursts increased with each training session; the rate of beta bursts increased in the final session. Relative power in the beta band (proposed marker of PD symptom severity) decreased over the motor cortex in the last session. Motor symptoms: changes in rigidity and freezing of gait were observed.	moderate
Cooke et al. (2024) (UK) *Neurophysiologie Clinique/Clinical Neurophysiology* [39]	Examine the comparative effects of PD medication versus EEG neurofeedback on central mu (9–11 Hz) power, handgrip test performance, and PD symptomatology. Test the feasibility and acceptability of EEG NF intervention in a home setting.	Experimental; within-subject design	Cortical activity: decreased central mu power Motor symptoms: Precision handgrip performance Motor Symptoms of PD: self-reported and observer-rated Non-motor symptoms: QoL Participant acceptability	n = 16 16 PD (10M/6F) *M*_age_ = 67.31 (SD = 9.77) H&Y 1−2; M years since diagnosis = 5.06	No control intervention	No	Cortical activity: mu power recorded during handgrip task performance was greater when OFF compared to ON medication during pre-tests. Mu power progressively decreased across three (OFF medication) EEG neurofeedback training sessions. Mu power at OFF medication post-test was similar to mu power recorded during ON medication pre-test. Neurofeedback was able to mimic the effects of medication on central mu power. Motor symptoms: linear improvements in handgrip movement planning time from pre-test to post-test and across the NF training sessions. Reduced variable error (pre-test to post-test); no significant changes in absolute error or constant error. EEG NF had no effect on objective (observer-rated) motor symptoms of PD; neither medication nor EEG NF resulted in a significant change in self-report measures related to motor aspects of daily living or quality of life. Acceptability: participants perceived some benefit of the EEG NF, improved walking, improved psychological control (e.g., ability to concentrate and relax), and improved motor control; nearly all participants (13/15) would recommend EEG NF to other people with PD.	low
Erickson-Davis et al. (2012) (USA) *Journal of Neurotherapy* [40]	Examine effects of EEG neurofeedback on cortical activity, levodopa-induced dyskinesia, and other clinical features of PD.	Placebo-controlled crossover RCT	Cortical activity: increased amplitude of a 3 Hz wide band within 8–15 Hz (alpha and low beta) with concurrent decreases in amplitude in both 4–8 Hz theta and 23–34 Hz high beta Motor symptoms: L-DOPA-induced dyskinesia and general motor symptoms	n = 9 Intervention: 5 PD (2M/3F) Control: 4 PD (2M/2F) *M*_age_ = 55.83 (SD = 11) H&Y 2.5 (SD = 0.35)	Sham followed by crossover EEG NF	No	Cortical activity: significant increases in alpha (8–12 Hz) and decreases in high beta (25–30 Hz) relative power post-intervention. Motor symptoms: no statistically significant differences in dyskinesia severity or in clinical features of PD. QoL: non-significant trends (home diaries) indicating a decrease in the severity of motor fluctuations.	moderate
Fumuro et al. (2013) (Japan) *Clinical Neurophysiology* [41]	Explore whether people with PD could increase readiness potential amplitude via EEG NF	Quasi-experimental; non-randomized controlled trial	Cortical activity: amplitude of the first and second components of the readiness potential—a negative-going waveform that occurs during preparation for movement Motor symptoms: not assessed	n = 21 Intervention: 10 PD (2M/8F) H&Y 3.5 (SD = 1.04) *M*_age_ =63.2 (SD = 11.45) Control: 11 age-matched healthy controls (1M/10F)	No control intervention (assessments of good NF vs. poor NF performance)	No	Cortical activity: Individual differences in responsiveness to the intervention within the intervention group. Intervention group participants were classified as good performers (slow cortical potentials differed between negative shift and positive shift training trials) or poor performers (no effect of training). Participants identified as good NF performers during the training phase appeared to learn to increase the amplitude of the early component of their readiness potential preceding a button press.	low
Han et al. (2023) (China) *IEEE* [42]	Explore whether EEG NF based on an imaginary movement strategy can regulate SMR relative power in people with PD	RCT	Cortical activity: SMR relative power Motor symptoms: mobility, balance, and risk of falling; motor aspects of experiences of daily living; and motor complications (dyskinesia and fluctuations) Non-motor symptoms: anxiety, depression, cognition, non-motor aspects of experiences of daily living	n = 14 Intervention: 7 PD (2M/5F) Control: 7PD (2M/5F) UPDRS III = 28.57 (SD = 10.7) *M*_age_ = 59.86 (SD = 8.44)	Sham EEG	No	Cortical activity: Cz SMR relative power increased significantly in the intervention group. Motor symptoms: Berg balance scale and timed up-and-go task performance improved from pre-test to post-test among members of the intervention group. No significant changes in other motor symptoms (e.g., UPDRS). Non-motor symptoms: No significant pre-test to post-test changes.	moderate
Kasahara et al. (2018) (Japan) *Brain-Computer Interfaces* [43]	Examine the effects of PD medication on the ability to modulate cortical activity via EEG neurofeedback. C3–C4 SMR (9.5–12.5 Hz) asymmetry in ON and OFF conditions	Case report	Cortical activity: C3–C4 SMR (9.5–12.5 Hz) asymmetry Motor symptoms: no target reported	n = 1 1 PD (1F) Age = 55 H&Y 2.5	No control intervention	No	Cortical activity: The participant was able to modulate their SMR activity via NF (success rate above chance level) while they were ON their PD medication. They were unable to regulate SMR power during the OFF medication test.	high
Legarda, Michas-Martin, and McDermott (2022) (USA) *Frontiers in Human Neuroscience* [44]	A case study to document the effects of infra-low frequency EEG NF on motor symptoms of PD	Case study	Cortical activity: increased delta and decreased beta spectral power Motor symptoms: tremor, writing skill, and gait	n = 3 3 PD (1M/2F) *M*_age_ =72 (SD = 6.4 years) disease severity not reported	No control intervention	No	Improved motor symptoms (tremor, dysgraphia, and freezing of gait) were observed	high
Romero et al. (2024) (Spain) *Journal of Neuroengineering and Rehabilitation* [45]	Determine immediate and short-term effects of EEG NF, alone or in combination with bilateral rTMS, in comparison to no intervention, in people with PD receiving pharmacologic therapy. Assess the electrophysiological correlates associated with the clinical effects	RCT	Cortical activity: Reduced central alpha (9–12 Hz) and beta (18–24 Hz) power Motor symptoms: severity, functional mobility, postural stability, and motor speed Non-motor symptoms: anxiety, depression, and QoL	n = 40 Intervention: arm A (rTMS): 10 PD (7M/3F) arm B (EEG NF): 11 PD (7M/4F) arm C (rTMS + EEG NF): 10 PD (7M/3F) Control: arm D (no intervention): 9 PD (5M/3F) H&Y 1.85 (SD = 0.50) UPDRS III = 15.8 (SD = 7.76) *M*_age_ = 63 (SD = 8.26)	rTMS; rTMS + EEG NF; control (no intervention)	Yes—15 days	Cortical activity: EEG data not reported. The EEG neurofeedback appeared to impact an adjacent non-EEG cortical marker—the cortical silent period—in the hypothesized way. Motor symptoms: rTMS alone improved motor symptoms. EEG NF alone has minimal direct impact on motor symptoms, although postural stability increased across assessments (all groups). The combination of rTMS and NF improved dominant hand motor skills. Non-motor symptoms and QoL: rTMS had no effect on depression; rTMS + EEG NF and EEG NF alone had small (negligible) effects.	moderate
Shi et al. (2023) (China) *Brain Science Advances* [46]	Explore whether biofeedback improves motor and non-motor functions in people with PD by regulating abnormal EEG, cardiac, pulse, respiration, or other physiological signals	RCT	Cortical activity: increased SMR relative power Motor symptoms: severity, balance, and fall risk Non-motor symptoms: behavior, mood, anxiety, and depression	n = 21 *M*_age_ = 59.86 (SD = 8.44) UPDRS III = 30.85 (SD = 11.15) Intervention: arm A (EEG NF) 7PD (2M/5F) arm B (multimodal EEG, ECG, PPG, and RSP) 7 PD (1M/6F) arm C Control group (sham EEG NF 7 PD (2M/5F)	Sham EEG NF	Yes—12 months	Cortical activity: Main effect—SMR relative power tended to be higher in the EEG NF and the multimodal biofeedback groups compared to the Sham group. Motor symptoms: Berg balance scale and timed up-and-go task performance improved from pre- to post-test among members of the EEG NF group. There were no effects on the other tests (e.g., UPDRS), and no effects emerged for the multimodal biofeedback group. Non-motor symptoms: No effects of EEG NF. Multimodal biofeedback was associated with reduced anxiety and depression.	moderate
Thompson and Thompson (2002) (Canada) *Journal of Neurotherapy* [21]	Present a theoretical framework for a biofeedback treatment for movement disorders in PD and dystonia	Case study	General motor symptoms	n = 1 1 (0M/1F) Age = 47 Years since diagnosis = 14	No control intervention	No	Reduction in dystonic movements by using diaphragmatic breathing to cue increased SMR production/self-reported control over freezing.	high

Note: Format adapted from the table preparation for Cochrane Public Health reviews: Overview of Synthesis and Included Studies table (OSIS) [26]. EEG NF: EEG neurofeedback training; PD: Parkinson’s disease; RCT: randomized controlled trial; SCP: slow cortical potential; RP: relative power; SMR: sensorimotor rhythm; rTMS: repetitive transcranial magnetic stimulation; ECG: electrocardiogram; PPG: photoplethysmography; and RSP: respiration.

**Table 3:** Summary of the neurofeedback intervention training protocols.

Study Reference	NF Targeted Activity/Activity Direction	NF Run Length	NF Session Length	Sessions	Total NF Exposure (Session Length × Sessions)	Time Between Sessions	Delivery Method	Instruction Given	Reported That Targeted EEG Activity Changed in the Prescribed Way
Azarpaikan, Torbati, and Sohrabi (2014) [37]	O1 and O2 Reinforce low β (12–15 Hz) activity (increase beta wave amplitude) and inhibit theta (4–7 Hz) activity (decrease theta wave amplitude)	Not reported	30 m	8	4 h	2–3 days	Video game, puzzle, and moving animation	Not reported	Yes
Cook, Pfeifer, and Tass (2021) [38]	C3 and C4 Increase SMR power (12–17 Hz) and high beta (17–30 Hz) activity over the motor cortex	5 m (10 blocks)	50 m	2	1 h 40 min	1 day	Visual feedback (constantly updating bar graph) + point system rewarding short increases in SMR activity.	Instructed to try to raise a bar graph on screen until it turns green and keep it green as long and often as possible	Yes
Cooke et al. (2024) [39]	C3 and C4—reduce mu power (9–11 Hz)	5 m (12 blocks)	1 h	3	3 h	Minimum 48 h; most common: 7 days	Audio feedback: power within the mu band from the EEG signal was fed back to participants in the form of an auditory tone; the tone was programmed to vary in pitch based on the level of mu power and silence completely when mu power was decreased by 30% (session 1), 55% (session 2), and 80% (session 3), relative to each participant’s baseline cortical activity	When thresholds were met, the auditory tone was set to silence for 1.5 s, and participants were instructed to squeeze the handgrip dynamometer with their dominant hand to produce a grip force equivalent to 10% MVC for 5 s (i.e., to initiate a trial of the precision motor task)	Yes
Erickson-Davis et al. (2012) [40]	C3 and C4 Reinforce alpha activity (8–15 Hz) and inhibit theta activity (4–8 Hz) and high beta (23–34 Hz) activity	Not reported	30 m	24	12 h	1–6 days	Audio feedback	No instruction	Yes
Fumuro et al. (2013) [41]	C3, C1, Cz, C2, and C4 Increase readiness potential amplitude (SCP negativation to restore decreased readiness potential)	10 s	8.7 m	2–4	18–36 min	1–6 days	Visual feedback of the subjects’ SCPs at Cz: sunfish moved up (negative shift/negativation) or down (positive shift/positivation) depending on the SCP shift	To make introspective efforts to produce negative SCPs (negativation)	Partial (individual differences in responsiveness to NF)
Han et al. (2023) [42]	C3 and C4 Increase SMR power	4 s	14 m	5	1 h 10 min	2 days	Visual: imaginary movement blocks; NF score was represented as a visual bar (height proportional to the score, color changing to reflect performance in relation to threshold)	To perform motion imagery according to the GIF animation played on a screen	Yes
Kasahara et al. (2018) [43]	C3 and C4 SMR (9.5–12.5 Hz) asymmetry	4 s	24 m	2 (on/off)	48 min	2 days	Visual: a falling cursor moved left or right to hit a target, depending on the targeted ERD (the signal used to control cursor movement was computed from the difference in the SMR amplitude between C4 and C3, where a greater ERD (decrease in power/amplitude) at C4 resulted in leftward online cursor movement and a greater ERD at C3 resulted in rightward cursor movement)	Motor imagery of the left or right hand: finger-thumb opposition to control the horizontal position of the cursor	Partial (EEG modulated ON medication only)
Legarda, Michas-Martin, and McDermott. (2022) [44]	T3-P3, T3-C3, and T3-F3; T4-P4, T4-C4, and T4-F4 Infra-low frequency NF expected to increase delta oscillation and suppress beta oscillation	Not reported	50 m	5	4 h 10 min	3 days	Not reported	Not reported	Only reported for one out of three cases: yes for the reported case
Romero et al. (2024) [45]	C3, Cz, and C4— reduce average bilateral alpha (9–12 Hz) and beta (18–24 Hz) bands Achieve beta desynchronization	10 s	30 m	8	4 h	1–2 days	Visual—virtual reality Move an object in 5 different 6-min virtual environments; each scenario had a virtual object that moved (feedback) when the average PSD was at least 1 SD lower than the average resting PSD in each frequency band; this threshold was adjusted daily to encourage progression in participant’s ability to self-regulate the signal.	No explicit instruction	EEG activity not reported. Modulation of a non-EEG brain-based measure (cortical silent period) was achieved, suggesting some impact of NF on the brain
Shi et al. (2023) [46]	C3 and C4 Increase SMR relative power	4 s	14 m	5	1 h 10 min	2 days	Visual: imaginary movement blocks; NF score was represented as a visual bar (height proportional to the score, color changing to reflect performance in relation to threshold)	To perform motion imagery according to the GIF animation played on a screen	Yes
Thompson and Thompson (2002) [21]	FCz and CPz Reinforce low beta/SMR (13–15 Hz) Inhibit alpha (6–10 Hz) activity Inhibit high beta (25–32 Hz) activity	Not reported	50 m	42	35 h	7 days	Visual and audio feedback (screen animations)	Not reported	Yes

SCP: slow cortical potential; RP: relative power; SMR: sensorimotor rhythm; PSD: power spectral density; and ERD: event-related desynchronization.

## Data Availability

The data presented were derived from the original articles cited in Table 2 and Table 3—the articles are already available in the public domain.

## Supplementary Materials

The following supporting information can be downloaded at: https://www.mdpi.com/article/10.3390/jcm14196929/s1, Figure S1: Risk of bias assessment Randomized Control Trials; Figure S2: Risk of bias assessment in non-randomized studies; Literature search strategy. Literature search strategy; PRISMA 2020 Checklist.

## References

[1] Clarke C.E. (2007). Parkinson’s disease. BMJ.

[2] Alty J.E., Clissold B.G., McColl C.D., Reardon K.A., Shiff M., Kempster P.A. (2009). Longitudinal study of the levodopa motor response in Parkinson’s disease: Relationship between cognitive decline and motor function. Mov. Disord..

[3] Pigott K., Rick J., Xie S.X., Hurtig H., Chen-Plotkin A., Duda J.E., Morley J.F., Chahine L.M., Dahodwala N., Akhtar R.S. (2015). Longitudinal study of normal cognition in Parkinson disease. Neurology.

[4] Latif S., Jahangeer M., Maknoon Razia D., Ashiq M., Ghaffar A., Akram M., El Allam A., Bouyahya A., Garipova L., Ali Shariati M. (2021). Dopamine in Parkinson’s disease. Clin. Chim. Acta.

[5] Muzerengi S., Clarke C.E. (2015). Initial drug treatment in Parkinson’s disease. BMJ.

[6] Lozano A.M., Dostrovsky J., Chen R., Ashby P. (2002). Deep brain stimulation for Parkinson’s disease: Disrupting the disruption. Lancet Neurol..

[7] Jankovic J., Aguilar L.G. (2008). Current approaches to the treatment of Parkinson’s disease. Neuropsych. Dis. Treat..

[8] Seijo F.J., Alvarez-Vega M.A., Gutierrez J.C., Fdez-Glez F., Lozano B. (2007). Complications in subthalamic nucleus stimulation surgery for treatment of Parkinson’s disease: Review of 272 procedures. Acta Neurochir..

[9] Hammond D.C. (2007). What is neurofeedback?. J. Neurother..

[10] Enriquez-Geppert S., Huster R.J., Herrmann C.S. (2017). EEG-neurofeedback as a tool to modulate cognition and behavior: A review tutorial. Front. Hum. Neurosci..

[11] Cheng M., Hung C., Huang C., Chang Y., Lo L., Shen C., Hung T. (2015). Expert-novice differences in SMR activity during dart throwing. Biol. Psychol..

[12] Gruzelier J.H. (2014). EEG-neurofeedback for optimising performance. II: Creativity, the performing arts and ecological validity. Neurosci. Biobehav. Rev..

[13] Ring C., Cooke A., Kavussanu M., McIntyre D., Masters R. (2015). Investigating the efficacy of neurofeedback training for expediting expertise and excellence in sport. Psychol. Sport Exerc..

[14] Mehler D.M. (2022). Turning markers into targets–scoping neural circuits for motor neurofeedback training in Parkinson’s disease. Brain Appar. Commun..

[15] Luctkar-Flude M., Groll D. (2015). A systematic review of the safety and effect of neurofeedback on fatigue and cognition. Integr. Cancer Ther..

[16] Devos D., Defebvre L. (2006). Effect of deep brain stimulation and L-Dopa on electrocortical rhythms related to movement in Parkinson’s disease. Prog. Brain Res..

[17] Dick J.P.R., Rothwell J.C., Day B.L., Cantello R., Buruma O., Gioux M., Benecke R., Berardelli A., Thompson P.D., Marsden C.D. (1989). The Bereitschaftspotential is abnormal in Parkinson’s disease. Brain.

[18] Leocani L., Comi G. (2006). Movement-related event-related desynchronization in neuropsychiatric disorders. Prog. Brain Res..

[19] Engel A.K., Fries P. (2010). Beta-band oscillations—Signalling the status quo?. Curr. Opin. Neurobiol..

[20] Jenkinson N., Brown P. (2011). New insights into the relationship between dopamine, beta oscillations and motor function. Trends Neurosci..

[21] Thompson M., Thompson L. (2002). Biofeedback for Movement Disorders (Dystonia with Parkinson’s Disease): Theory and Preliminary Results. J. Neurother..

[22] Esmail F., Linden D.E.J. (2014). Neural Networks and Neurofeedback in Parkinson’s Disease. NeuroRegulation.

[23] Anil K., Hall S.D., Demain S., Freeman J.A., Ganis G., Marsden J. (2021). A systematic review of neurofeedback for the management of motor symptoms in Parkinson’s disease. Brain Sci..

[24] Blaznik L., Marusic U. (2025). Exploring the Impact of Electroencephalography-Based Neurofeedback (EEG NFB) on Motor Deficits in Parkinson’s Disease: A Targeted Literature Review. Appl. Sci..

[25] Page M.J., McKenzie J.E., Bossuyt P.M., Boutron I., Hoffmann T.C., Mulrow C.D., Shamseer L., Tetzlaff J.M., Akl E.A., Brennan S.E. (2021). The PRISMA 2020 statement: An updated guideline for reporting systematic reviews. BMJ.

[26] Higgins J., Thomas J., Chandler J., Cumpston M., Li T., Page M.J., Welch V.A. (2024). Cochrane Handbook for Systematic Reviews of Interventions.

[27] Postuma R.B., Berg D., Stern M., Poewe W., Olanow C.W., Oertel W., Obeso J., Marek K., Litvan I., Lang A.E. (2015). MDS clinical diagnostic criteria for Parkinson’s disease. Mov. Disord..

[28] Greenhalgh T., Peacock R. (2005). Effectiveness and efficiency of search methods in systematic reviews of complex evidence: Audit of primary sources. BMJ.

[29] Ouzzani M., Hammady H., Fedorowicz Z., Elmagarmid A. (2016). Rayyan—A web and mobile app for systematic reviews. Syst. Rev..

[30] Sterne J.A.C., Savović J., Page M.J., Elbers R.G., Blencowe N.S., Boutron I., Cates C.J., Cheng H., Corbett M.S., Eldridge S.M. (2019). RoB 2: A revised tool for assessing risk of bias in randomised trials. BMJ.

[31] Sterne J., Hernán M.A., Reeves B.C., Savović J., Berkman N.D., Viswanathan M., Henry D., Altman D.G., Ansari M.T., Boutron I. (2016). ROBINS-I: A tool for assessing risk of bias in non-randomised studies of interventions. BMJ.

[32] McGuinness L.A., Higgins J.P.T. (2020). Risk-of-bias VISualization (robvis): An R package and Shiny web app for visualizing risk-of-bias assessments. Res. Synth. Methods.

[33] Goetz C.G., Tilley B.C., Shaftman S.R., Stebbins G.T., Fahn S., Martinez-Martin P., Poewe W., Sampaio C., Stern M.B., Dodel R. (2008). Movement Disorder Society-sponsored revision of the Unified Parkinson’s Disease Rating Scale (MDS-UPDRS): Scale presentation and clinimetric testing results. Mov. Disord..

[34] Cohen J. (1992). A power primer. Psychol. Bull..

[35] Guyatt G.H., Oxman A.D., Vist G.E., Kunz R., Falck-Ytter Y., Alonso-Coello P., Schünemann H.J. (2008). GRADE: An emerging consensus on rating quality of evidence and strength of recommendations. BMJ.

[36] Egger M., Smith G.D., Schneider M., Minder C. (1997). Bias in meta-analysis detected by a simple, graphical test. BMJ.

[37] Azarpaikan A., Torbati H.T., Sohrabi M. (2014). Neurofeedback and physical balance in Parkinson’s patients. Gait Posture.

[38] Cook A.J., Pfeifer K.J., Tass P.A. (2021). A single case feasibility study of sensorimotor rhythm neurofeedback in Parkinson’s disease. Front. Neurosci..

[39] Cooke A., Hindle J., Lawrence C., Bellomo E., Pritchard A.W., MacLeod C.A., Martin-Forbes P., Jones S., Bracewell M., Linden D.E.J. (2024). Effects of home-based EEG neurofeedback training as a non-pharmacological intervention for Parkinson’s disease. Neurophysiol. Clin..

[40] Erickson-Davis C., Anderson J.S., Wielinski C.L., Richter S.A., Parashos S.A. (2012). Evaluation of neurofeedback training in the treatment of Parkinson’s disease: A pilot study. J. Neurother..

[41] Fumuro T., Matsuhashi M., Mitsueda T., Inouchi M., Hitomi T., Nakagawa T., Matsumoto R., Kawamata J., Inoue H., Mima T. (2013). Bereitschaftspotential augmentation by neuro-feedback training in Parkinson’s disease. Clin. Neurophysiol..

[42] Han X., Shi Z., Pei G., Fang B., Yan T. Effect of neurofeedback based on imaginary movement in Parkinson’s disease. Proceedings of the 2023 17th International Conference on Complex Medical Engineering (CME).

[43] Kasahara K., Hoshino H., Furusawa Y., DaSalla C.S., Honda M., Murata M., Hanakawa T. (2018). Initial experience with a sensorimotor rhythm-based brain-computer interface in a Parkinson’s disease patient. Brain Comput. Interfaces.

[44] Legarda S.B., Michas-Martin P.A., McDermott D. (2022). Managing intractable symptoms of Parkinson’s disease: A nonsurgical approach employing infralow frequency neuromodulation. Front. Hum. Neurosci..

[45] Romero J.P., Moreno-Verdú M., Arroyo-Ferrer A., Serrano J.I., Herreros-Rodríguez J., García-Caldentey J., de Lima E.R., del Castillo M.D. (2024). Clinical and neurophysiological effects of bilateral repetitive transcranial magnetic stimulation and EEG-guided neurofeedback in Parkinson’s disease: A randomized, four-arm controlled trial. J. Neuroeng. Rehabil..

[46] Shi D., Han J., Zhang S., Suo W., Wu P., Pei F., Fang B., Yan T. (2023). Multimodal biofeedback for Parkinson’s disease motor and nonmotor symptoms. Brain Sci. Adv..

[47] Chen T.T., Wang K.P., Chang W.H., Kao C.W., Hung T.M. (2022). Effects of the function-specific instruction approach to neurofeedback training on frontal midline theta waves and golf putting performance. Psychol. Sport Exerc..

[48] Wang H.C., Lees A.J., Brown P. (1999). Impairment of EEG desynchronisation before and during movement and its relation to bradykinesia in Parkinson’s disease. J. Neurol. Neurosurg. Psychiatry.

[49] Kühn A.A., Doyle L., Pogosyan A., Yarrow K., Kupsch A., Schneider G.H., Hariz M.I., Trottenberg T., Brown P. (2006). Modulation of beta oscillations in the subthalamic area during motor imagery in Parkinson’s disease. Brain.

[50] Linden D.E. (2014). Neurofeedback and networks of depression. Dialogues Clin. Neurosci..

[51] Poon S.F., Tan C.H., Hong W.P., Chen K.C., Yu R.L. (2025). Tailoring anxiety assessment for Parkinson’s disease: The Chinese Parkinson anxiety scale with cultural and situational anxiety considerations. Soc. Sci. Med..

[52] Yi H.J., Tan C.H., Hong W.P., Yu R.L. (2024). Development and validation of the geriatric apathy scale: Examining multi-dimensional apathy profiles in a neurodegenerative population with cultural considerations. Asian J. Psychiatry.

[53] Moher D., Schulz K.F., Altman D.G. (2001). The CONSORT statement: Revised recommendations for improving the quality of reports of parallel-group randomised trials. Lancet.

