# Supplementary material for: Effectiveness of Electroencephalographic Neurofeedback for Parkinson’s Disease: A Systematic Review and Meta-Analysis
Source: J Clin Med. 2025 Sep 30;14(19):6929. doi: 10.3390/jcm14196929 (PMC12524379; doi:10.3390/jcm14196929)
Supplement: Supplementary file 1 [file jcm-14-06929-s001.zip › jcm-3776361-supplementary.pdf]

**Literature search strategy**

**Search methods for study identification**

The search strategy comprised the major healthcare electronic databases: Cochrane Database of Systematic Reviews, Cochrane Database of Abstracts of Effectiveness, CENTRAL via The Cochrane Register of Studies, PubMed, Embase, Scopus, Web of Science Core Collection, and PsycInfo.
In addition, the search included the most common trial registers, such as the WHO International Clinical Trials Registry Platform, ClinicalTrials.gov, EUDRACT, and ISRCTN; the "UK National Research Register" (discontinued and archived in 2021 but remains a useful repository for records held in the National Archives), was also searched; the "System for Information on Grey Literature" was searched for bibliographical references or doctoral dissertations, conference papers, research reports, and other types of grey literature; the 'Research Registry', a IJS/Wolters Kluwer resource; the NIHR "Be Part of Research" and the HRA Research Summaries for ongoing studies that have not yet reached the publication stage but may have interim results.
'Lateral searching' techniques such as checking reference lists and tracking citations were used as recommended by Greenhalgh and Peacock (2005).
The inclusion criteria comprised the following: Human population; Sample age (adults); Methodological approach (experimental/interventional, quantitative and qualitative studies); Publication date from 2014 to 2024; Methodological rigour: peer reviewed studies published in indexed journals; Language: English, (although articles published in French and German were also retrieved)
Three separate searches were run, with the help of a specialist librarian from Bangor University, the latest one on 24 October 2024. A list of keywords and the search strategies used are presented below.

**PubMed**

Search: (("Neurofeedback"[Mesh]) OR (Brainwave Biofeedback[Title/Abstract] OR EEG Feedback[Title/Abstract] OR Electroencephalography Biofeedback[Title/Abstract] OR Brainwave Feedback[Title/Abstract]) AND ((y_10[Filter]) AND (english[Filter]))) AND ((Parkinson's Disease[Title/Abstract] OR PD[Title/Abstract] OR Parkinsonism[Title/Abstract] AND ((y_10[Filter]) AND (english[Filter]))) OR ("Parkinson Disease"[Mesh]) AND ((y_10[Filter]) AND (humans[Filter]) AND (english[Filter]))) - Saved search Filters: in the last 10 years, Humans, English Sort by: Most Recent
(("Neurofeedback"[MeSH Terms] OR ("brainwave biofeedback"[Title/Abstract] OR "eeg feedback"[Title/Abstract] OR "electroencephalography biofeedback"[Title/Abstract] OR (("brain waves"[MeSH Terms] OR ("brain"[All Fields] AND "waves"[All Fields]) OR "brain waves"[All Fields] OR "Brainwave"[All Fields] OR "brainwaves"[All Fields]) AND "Feedback"[Title/Abstract]))) AND ("2014/10/29 00:00":"3000/01/01 05:00"[Date - Publication] AND "english"[Language]) AND (((("parkinson s disease"[Title/Abstract] OR "PD"[Title/Abstract] OR "Parkinsonism"[Title/Abstract]) AND ("2014/10/29 00:00":"3000/01/01 05:00"[Date - Publication] AND "english"[Language])) OR "Parkinson Disease"[MeSH Terms]) AND ("2014/10/29 00:00":"3000/01/01 05:00"[Date - Publication] AND "humans"[MeSH Terms] AND "english"[Language]))) AND ((y_10[Filter]) AND (humans[Filter]) AND (english[Filter])) Translations
Brainwave: "brain waves"[MeSH Terms] OR ("brain"[All Fields] AND "waves"[All Fields]) OR "brain waves"[All Fields] OR "brainwave"[All Fields] OR "brainwaves"[All Fields]
y_10[Filter]: "last 10 years"[dp] english[Filter]: english [LA] y_10[Filter]: "last 10 years"[dp] english[Filter]: english [LA] y_10[Filter]: "last 10 years"[dp] humans[Filter]: humans[MH] english[Filter]: english [LA]

**Web of Science**

EEG Neurofeedback OR Brainwave Biofeedback OR EEG Feedback OR Electroencephalography Biofeedback OR Brainwave Feedback (Title) and Parkinson's Disease OR PD OR Parkinsonism OR Parkinson* (All Fields)
EEG Neurofeedback OR Brainwave Biofeedback OR EEG Feedback OR Electroencephalography Biofeedback OR Brainwave Feedback (Topic) and Essential Tremor OR bilateral upper limb tremor (All Fields)

**Embase - Embase, MEDLINE, Preprints**
Query(((((((('eeg'/exp OR eeg) AND ('neurofeedback'/exp OR neurofeedback) OR 'brainwave'/exp OR brainwave) AND ('biofeedback'/exp OR biofeedback) OR 'brainwave'/exp OR brainwave) AND ('biofeedback'/exp OR biofeedback) OR 'eeg'/exp OR eeg) AND ('feedback'/exp OR feedback) OR 'eeg'/exp OR eeg) AND ('feedback'/exp OR feedback) OR 'electroencephalography'/exp OR electroencephalography) AND ('biofeedback'/exp OR biofeedback) OR 'electroencephalography'/exp OR electroencephalography) AND ('biofeedback'/exp OR biofeedback) OR 'brainwave'/exp OR brainwave) AND ('feedback'/exp OR feedback) AND ((parkinsons AND 'disease'/de OR 'parkinsonism'/de OR parkinson) AND 'disease'/de OR 'parkinson disease'/de) AND [english]/lim AND [humans]/lim AND [2014-2024]/py AND ([article]/lim OR [article in press]/lim OR [editorial]/lim OR [review]/lim)

**PsycInfo**
(MAINSUBJECT.EXACT.EXPLODE("Parkinson's Disease") OR MAINSUBJECT.EXACT("Parkinsonism") OR MAINSUBJECT.EXACT("Parkinson's Disease")) AND (MAINSUBJECT.EXACT("Biofeedback") OR MAINSUBJECT.EXACT("Biofeedback Training") OR MAINSUBJECT.EXACT("Feedback") OR MAINSUBJECT.EXACT("Neurotherapy")) AND la.exact("English") AND po.exact("human") AND pd(>20131231)
(MAINSUBJECT.EXACT.EXPLODE("Parkinson's Disease") OR MAINSUBJECT.EXACT("Parkinsonism") OR MAINSUBJECT.EXACT("Parkinson's Disease")) AND MAINSUBJECT.EXACT (“EEG Neurofeedback”) OR MAINSUBJECT.EXACT (“Brainwave Biofeedback”) OR MAINSUBJECT.EXACT (“EEG feedback”) OR MAINSUBJECT.EXACT (“Electroencephalography Biofeedback”) OR MAINSUBJECT.EXACT (“Brainwave feedback”) OR MAINSUBJECT.EXACT("Biofeedback") OR MAINSUBJECT.EXACT("Biofeedback Training") OR MAINSUBJECT.EXACT("Feedback") OR MAINSUBJECT.EXACT("Neurotherapy")) AND la.exact("English") AND po.exact("human") AND pd(>20131231)

**Scopus**
TITLE-ABS-KEY (("EEG Neurofeedback") OR ("Brainwave Biofeedback") OR ("EEG Feedback") OR ("Electroencephalography Biofeedback") OR ("Brainwave Feedback")) AND TITLE-ABS-KEY ((" Parkinson’s Disease") OR ("PD") OR ("Parkinsonism"))

**Clinicaltrials.gov**
**Advanced search**
Conditions: Parkinson; Interventions: EEG neurofeedback OR biofeedback OR train* OR intervention*; Recruitment: All studies; Study type: Interventional studies

**WHO ICTRP**
**Advanced search**
Condition: Parkinson*
Intervention: EEG neurofeedback OR biofeedback* OR movement OR train* OR mind body OR therap*; Recruitment status: ALL

**ISRCTN search strategy:**
Condition: Parkinson

**EU Clinical Trials Register search strategy:**
Parkinson; Neurofeedback

# Risk of Bias Assessment

**Figure S1** Risk of bias assessment Randomized Control Trials

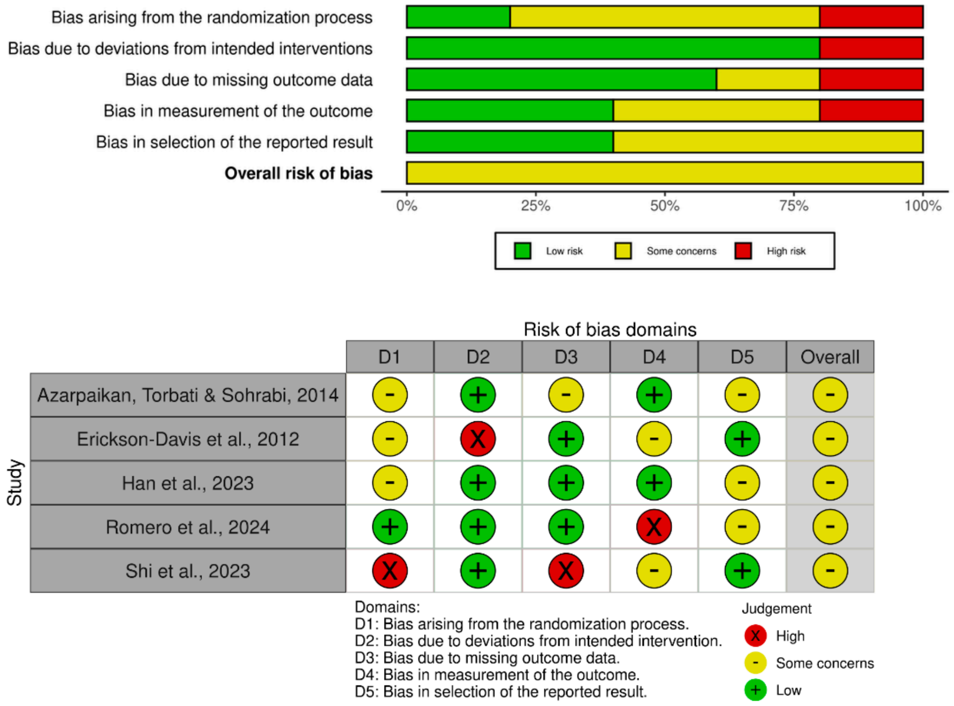


*Note:* The Risk of Bias Assessment was conducted using the RoB 2 tool—Cochrane Risk of Bias in randomized trials (Sterne et al., 2019) https://methods.cochrane.org/bias/resources/rob-2-revised-cochrane-risk-bias-tool-randomized-trials, accessed 27 November 2024

**Figure S2** Risk of bias assessment in non-randomized studies

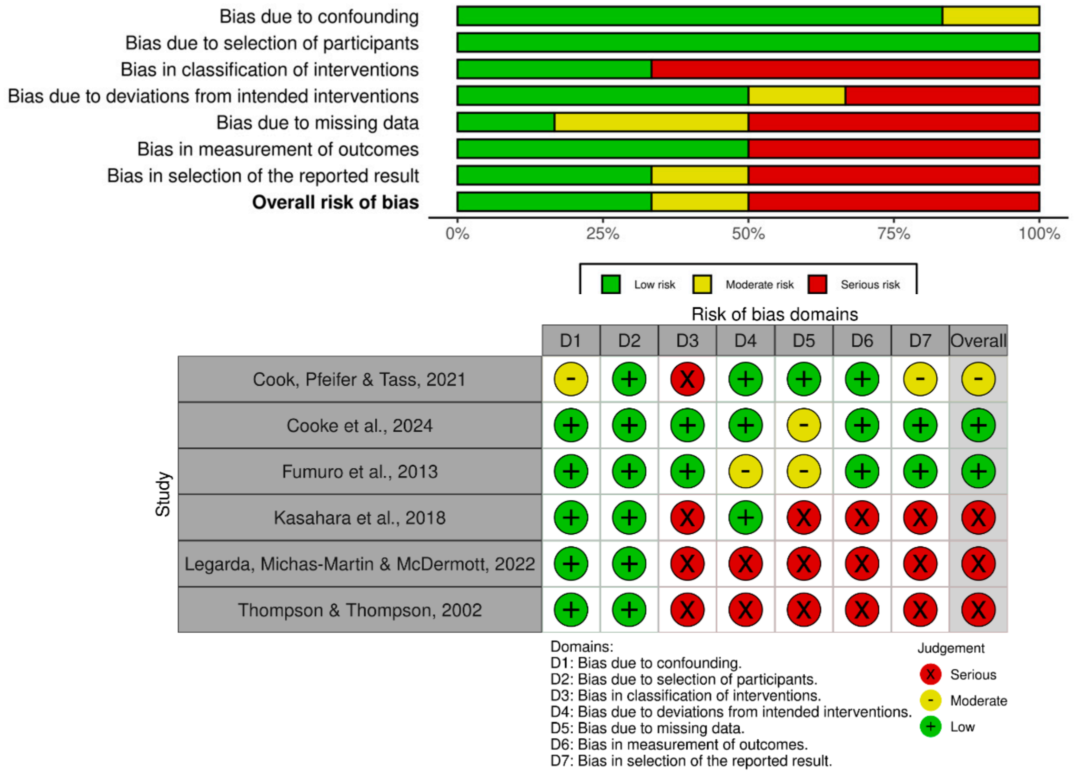


*Note:* The Risk of Bias Assessment was conducted using the Risk Of Bias In Non-Randomized Studies - of Interventions (ROBINS-I) (Sterne et al., 2016) https://methods.cochrane.org/bias/risk-bias-non-randomized-studies-interventions, accessed 30 November 2024

# PRISMA 2020 Checklist

| Section and Topic | Item # | Checklist item | Location where item is reported |
|---|---|---|---|
| **TITLE** | | | |
| Title | 1 | Identify the report as a systematic review. | Title |
| **ABSTRACT** | | | |
| Abstract | 2 | See the PRISMA 2020 for Abstracts checklist. | Abstract |
| **INTRODUCTION** | | | |
| Rationale | 3 | Describe the rationale for the review in the context of existing knowledge. | Introduction (1) |
| Objectives | 4 | Provide an explicit statement of the objective(s) or question(s) the review addresses. | Introduction (1.3, 1.4, 1.5) |
| **METHODS** | | | |
| Eligibility criteria | 5 | Specify the inclusion and exclusion criteria for the review and how studies were grouped for the syntheses. | Selection criteria (2.1) & Table 1. |
| Information sources | 6 | Specify all databases, registers, websites, organisations, reference lists and other sources searched or consulted to identify studies. Specify the date when each source was last searched or consulted. | Search Strategy (2.2) |
| Search strategy | 7 | Present the full search strategies for all databases, registers and websites, including any filters and limits used. | Search Strategy (p. 4) and Supplementary Online Material |
| Selection process | 8 | Specify the methods used to decide whether a study met the inclusion criteria of the review, including how many reviewers screened each record and each report retrieved, whether they worked independently, and if applicable, details of automation tools used in the process. | Study Selection (2.3) & Data Extraction and Management (2.4) |
| Data collection process | 9 | Specify the methods used to collect data from reports, including how many reviewers collected data from each report, whether they worked independently, any processes for obtaining or confirming data from study investigators, and if applicable, details of automation tools used in the process. | Data Extraction and Management (p. 5) |
| Data items | 10a | List and define all outcomes for which data were sought. Specify whether all results that were compatible with each outcome domain in each study were sought (e.g. for all measures, time points, analyses), and if not, the methods used to decide which results to collect. | Data Extraction and Management (2.4) |
| | 10b | List and define all other variables for which data were sought (e.g. participant and intervention characteristics, funding sources). Describe any assumptions made about any missing or unclear information. | Data Extraction and Management |

| Section and Topic | Item # | Checklist item | Location where item is reported |
|---|---|---|---|
| | | | (2.4) |
| Study risk of bias assessment | 11 | Specify the methods used to assess risk of bias in the included studies, including details of the tool(s) used, how many reviewers assessed each study and whether they worked independently, and if applicable, details of automation tools used in the process. | Risk of Bias Assessment (2.5) |
| Effect measures | 12 | Specify for each outcome the effect measure(s) (e.g. risk ratio, mean difference) used in the synthesis or presentation of results. | Meta-analysis (2.6) |
| Synthesis methods | 13a | Describe the processes used to decide which studies were eligible for each synthesis (e.g. tabulating the study intervention characteristics and comparing against the planned groups for each synthesis (item #5)). | Data Extraction and Management (2.5) & Tables 2 & 3 |
| | 13b | Describe any methods required to prepare the data for presentation or synthesis, such as handling of missing summary statistics, or data conversions. | Meta-analysis (2.6) |
| | 13c | Describe any methods used to tabulate or visually display results of individual studies and syntheses. | Data Extraction and Management (2.5). Meta-analysis (2.6). Tables 2 & 3. |
| | 13d | Describe any methods used to synthesize results and provide a rationale for the choice(s). If meta-analysis was performed, describe the model(s), method(s) to identify the presence and extent of statistical heterogeneity, and software package(s) used. | Meta-analysis (2.6). Assessment of Reporting Bias (2.8). Included Studies (3.2) |
| | 13e | Describe any methods used to explore possible causes of heterogeneity among study results (e.g. subgroup analysis, meta-regression). | Meta-analysis (2.6). Subgroup analyses (2.7). Assessment of Reporting Bias (2.8). |
| | 13f | Describe any sensitivity analyses conducted to assess robustness of the synthesized results. | Meta-analysis (2.6). Assessment of Reporting Bias (2.8). |

| Section and Topic | Item # | Checklist item | Location where item is reported |
|---|---|---|---|
| Reporting bias assessment | 14 | Describe any methods used to assess risk of bias due to missing results in a synthesis (arising from reporting biases). | Assessment of Reporting Bias (2.8). |
| Certainty assessment | 15 | Describe any methods used to assess certainty (or confidence) in the body of evidence for an outcome. | Assessment of Reporting Bias (2.8). |
| **RESULTS** | | | |
| Study selection | 16a | Describe the results of the search and selection process, from the number of records identified in the search to the number of studies included in the review, ideally using a flow diagram. | Search results (3.1) & Figure 1 |
| | 16b | Cite studies that might appear to meet the inclusion criteria, but which were excluded, and explain why they were excluded. | Effects of EEG neurofeedback on cortical activity (3.5). |
| Study characteristics | 17 | Cite each included study and present its characteristics. | Tables 2 & 3. |
| Risk of bias in studies | 18 | Present assessments of risk of bias for each included study. | Figure 2 and Supplementary Material |
| Results of individual studies | 19 | For all outcomes, present, for each study: (a) summary statistics for each group (where appropriate) and (b) an effect estimate and its precision (e.g. confidence/credible interval), ideally using structured tables or plots. | Tables 2 & 3, Figures 3 & 4. |
| Results of syntheses | 20a | For each synthesis, briefly summarise the characteristics and risk of bias among contributing studies. | Study Quality Appraisal (3.3) |
| | 20b | Present results of all statistical syntheses conducted. If meta-analysis was done, present for each the summary estimate and its precision (e.g. confidence/credible interval) and measures of statistical heterogeneity. If comparing groups, describe the direction of the effect. | Meta-analyses (3.4) |
| | 20c | Present results of all investigations of possible causes of heterogeneity among study results. | Meta-analyses (3.4)<br>Tables 2 & 3<br>Discussion (4). |
| | 20d | Present results of all sensitivity analyses conducted to assess the robustness of the synthesized results. | Study Quality Appraisal (3.3)<br>Meta-analyses (3.4)<br>Tables 2 & 3 |
| Reporting biases | 21 | Present assessments of risk of bias due to missing results (arising from reporting biases) for each synthesis assessed. | Study Quality Appraisal (3.3)<br>Meta-analyses (3.4) |

| Section and Topic | Item # | Checklist item | Location where item is reported |
|---|---|---|---|
| | | | Tables 2 & 3 |
| Certainty of evidence | 22 | Present assessments of certainty (or confidence) in the body of evidence for each outcome assessed. | Study Quality Appraisal (3.4) Meta-analyses (3.4) Tables 2 & 3 |
| **DISCUSSION** | | | |
| Discussion | 23a | Provide a general interpretation of the results in the context of other evidence. | Discussion (4) |
| | 23b | Discuss any limitations of the evidence included in the review. | Discussion (4) |
| | 23c | Discuss any limitations of the review processes used. | Discussion (4) |
| | 23d | Discuss implications of the results for practice, policy, and future research. | Discussion (4.5) |
| **OTHER INFORMATION** | | | |
| Registration and protocol | 24a | Provide registration information for the review, including register name and registration number, or state that the review was not registered. | Materials & Methods (2) |
| | 24b | Indicate where the review protocol can be accessed, or state that a protocol was not prepared. | Materials & Methods (2) |
| | 24c | Describe and explain any amendments to information provided at registration or in the protocol. | n/a |
| Support | 25 | Describe sources of financial or non-financial support for the review, and the role of the funders or sponsors in the review. | Funding (author notes) |
| Competing interests | 26 | Declare any competing interests of review authors. | Conflicts of Interest (author notes) |
| Availability of data, code and other materials | 27 | Report which of the following are publicly available and where they can be found: template data collection forms; data extracted from included studies; data used for all analyses; analytic code; any other materials used in the review. | Data Availability Statement (author notes) and Supplementary Online Material |

*From:* Page MJ, McKenzie JE, Bossuyt PM, Boutron I, Hoffmann TC, Mulrow CD, et al. The PRISMA 2020 statement: an updated guideline for reporting systematic reviews. BMJ 2021;372:n71. doi: 10.1136/bmj.n71

For more information, visit: http://www.prisma-statement.org/
